# Isothermal Kinetics of Epoxyphosphazene Cure

**DOI:** 10.3390/polym13020297

**Published:** 2021-01-18

**Authors:** Natalia V. Bornosuz, Irina Yu. Gorbunova, Viktoria V. Petrakova, Vyacheslav V. Shutov, Vyacheslav V. Kireev, Denis V. Onuchin, Igor S. Sirotin

**Affiliations:** Faculty of Petrochemistry and Polymer Materials, Mendeleev University of Chemical Technology of Russia, Miusskaya sq. 9, 125047 Moscow, Russia; bornosuz@muctr.ru (N.V.B.); igorbunova@muctr.ru (I.Y.G.); vvvorobeva@muctr.ru (V.V.P.); shutov1105@gmail.com (V.V.S.); kireev@muctr.ru (V.V.K.); donuchin@muctr.ru (D.V.O.)

**Keywords:** epoxy resins, epoxyphosphazenes, kinetics of curing, isoconversional kinetics, model fitting

## Abstract

The influence of epoxycyclophosphazene modifier on the process of epoxy-amine curing was studied by differential scanning calorimetry (DSC). The study revealed that the curing process of epoxyphosphazene binders with 4′4′diaminodiphenylsulfone (DDS) provides more complete curing of the formulations in comparison with ones applying low molecular-weight polyamide curing agent (L-20). The isothermal kinetics of curing was described by means of model fitting and the isoconversional approach (Friedman method). Accurate n-order approximation was obtained for all systems under study. In particular, the 2-order equation fits well with the main part of curing excluding high degrees of conversion. The process of curing could be distinguished into three zones. The transition from zone 2 to zone 3 correlates with gelation. According to the isoconversional analysis by Friedman method, the diffusion-controlled mechanism is found at final stage of curing.

## 1. Introduction

Epoxy binders for polymer composites play an important role in modern high-tech materials and are indispensable in the aerospace and transport industries. Epoxy polymers are non-competitive in terms of their mechanical properties, lightness and price compared to the other classes of thermosetting polymers [1]. At the same time, their high flammability excludes the use of epoxy resins for materials with high fire resistance requirements, for example for cabin interior trim [2,3]. For this purpose coke-forming phenolic or polybenzoxazine matrices [1] or epoxy and polyester binders modified with flame retardants are usually used [2,4]. However, the introduction, for instance, of phosphorus-containing flame retardants, as a rule, leads to a significant decrease in mechanical properties and heat resistance of the polymer due to the absence of a covalent bond between matrix and flame retardant resulting in two-phase system. Thus, they are not widely used in critical parts.

A promising way to solve the problem of the flammability of epoxy polymers is the use of modifiers based on phosphazenes containing reactive epoxy groups in the organic radical and, due to the latter, forming a covalent bond with the matrix during curing. The main chain of organophosphazenes consists of alternating phosphorus and nitrogen atoms, and at the phosphorus atom there are organic radicals introduced by the substitution of halogens in halogen phosphazenes. The nature of organic substituents can usually vary widely, and this diversity determines the properties of the final polymer or oligomer. Different types of product, whether resins, flame retardants or curing agents that can be easily obtained on the basis of universal halophosphazenes, are responsible for the undying interest of researchers in phosphazenes [5,6,7,8,9,10,11,12]. Arylphosphazenes compared with other organophosphorus compounds, have, as a rule, higher thermal stability and chemical resistance and are promising fire retardants [4,6,13,14], characterized by the synergistic effect of phosphorus and nitrogen [15]. Thus, phenoxycyclophosphazenes were commercialized as a flame retardant by Otsuka Chemical et al. Recently it was shown that epoxy-amine matrices modified with epoxycyclophosphazene [16] are characterized by enhanced mechanical properties [17]. Therefore, epoxy resins modified with epoxycyclophosphazenes appear to be a promising base for halogen-free binders for critical use. Recently, scalable methods for epoxycyclophosphazene synthesis have appeared [6,16,18], which make it possible to believe that they may become available starting components in the market for composite manufacture.

A considerable part of the mechanical properties of thermosets is determined by a three-dimensional network formed during curing. So the study of the curing kinetics is the next step for the proper predictions. Kinetics models are applied while developing a cure mode and calculating for warpage, for example.

This work is dedicated to the study of the isothermal curing of the epoxy-amine matrix modified with epoxycyclophosphazene [16]. In particular, the modifier influence on the main kinetic parameters of the curing process is revealed.

The methods of kinetic study are divided into two large groups: the model-free and the model fitting approach. The main advantage of model-free (isoconversional) approach is the independence of any kinetic equation approximation. This approach makes it possible to estimate the kinetic parameters of the process in time, especially the dependence of effective activation energy on the degree of conversion that is then usually used as the initial approximation when constructing an accurate kinetic model [19]. However, there are some disadvantages of this approach. The explicit determination of the kinetic equation type is impossible and among all kinetic parameters, only the activation energy and preexponential factor are uniquely determined.

The model fitting approach allows us to analyze complex multistage reactions. The dependencies can be approximated by well-known kinetic equations. In the case of application, it is possible to create quite an accurate model of the process using non-linear regression methods.

The differential scanning calorimetry (DSC) method is the most widespread method for monitoring the curing process [20]. It is based on the measurement of the heat flow, which is proportional to both overall heat release and the rate of reaction [21].

(1) $$\frac{dQ}{d\tau }=Q_{max}\cdot \frac{d\beta }{d\tau }=Q_{max}\cdot K\left(T\right)\cdot f(\beta )$$

where $\frac{dQ}{d\tau }$ is the heat flow, $\frac{d\beta }{d\tau }$ the cure rate, K(T) the rate constant, f(β) the reaction model, $Q_{max}$ the total heat released during the complete curing process.

A large number of studies have shown that when curing epoxy oligomers f(β) usually has the form (1 − *β*)^n^, where *n* is the reaction order, or *β*^m^(1 − *β*)^n^ in the case of autocatalytic reaction [22,23]. The temperature dependence of the rate constant is introduced by replacing *K(T)* with the Arrhenius equation:

(2) $$\frac{d\beta }{d\tau }=A\cdot \exp\left(-\frac{E}{RT}\right)\cdot f(\beta )$$

where *A* is the pre-exponential factor, *E* the effective activation energy and *R* is the gas constant.

Today researchers can utilize a wide range of both model fitting and isoconversional model-free methods applicable for isothermal and non-isothermal experiments. Among the numerous isoconversional methods applicable for non-isothermal modes [24,25,26,27], some of them, for example the Fridman method [25] and the Vyazovkin method [21] are applicable to the process of isothermal curing.

## 2. Materials and Methods

### 2.1. Materials

We studied the effect of epoxycyclophosphazene on the kinetic parameters during isothermal curing of the binder based on bisphenol-A-based epoxy resin (ED-20) with a hardener 4′4′diaminodiphenylsulfone (DDS).

Bifunctional bisphenol-A-based epoxy resin of ED-20 brand (satisfied the Russian standard GOST 10587-84), manufactured at the Y.M. Sverdlov plant (Dzerzhinsk, Russia), Mn = 390 g/mol, epoxy group content of 20.0–22.0%, and dynamic viscosity of 18.4 Pa·s at 25 °C, were used as the epoxy resin matrix.

**Figure 1:**
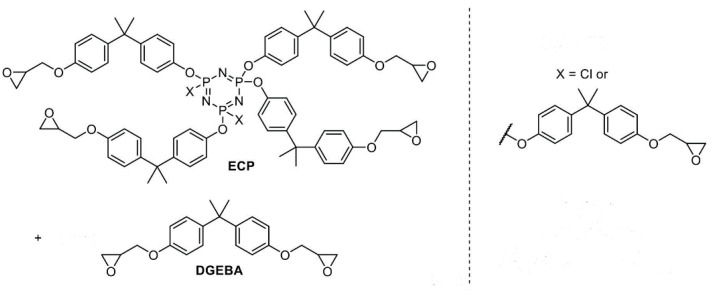
The general formula of epoxycyclophosphazene resin (products of the reaction of epichlorohydrin with hydroxyaryloxycyclotriphosphazenes). Epoxycyclophosphazene oligomer (ECP), diglycidyl ether of bisphenol A (DGEBA).

An epoxypcyclohosphazene resin, which is a homogeneous mixture of a conventional bisphenol-A-based epoxy oligomer (DGEBA) and epoxycyclophosphazene oligomer (ECP) of the following general formula (Figure 1), was obtained at the Mendeleev University of Chemical Technology (Moscow, Russia) according to the technique [18] in such a way that the ECP content in the epoxyphosphazene resin was 50% by weight. Epoxyphosphazene modifier is a high-viscosity liquid with a dynamic viscosity of 100–200 Pa⋅s at 40 °C [28], a mean value of M_w_ is 850 g/mol, the molecular weight ECP component is 1000–1800 g/mol, and the content of epoxy groups in the mixture is 17–19%, chlorine is 1.5–2.0%.

ECP was mixed with epoxy resin ED-20 at 80 °C so that the content of ECP in the epoxy component of the binder varied from 0 and 20 wt.%.

As the curing agent, 4′4′diaminodiphenylsulfone (DDS) of the Aradur 9664-1 brand from Huntsman (Munich, Germany) was used in the form of a fine powder with a particle size of less than 64 μm and a Tm = 175 °C. It was added to the above-mentioned mixtures in a stoichiometric ratio.

The studied compositions are presented in Table 1.

The calculated amount of ED-20 and epoxyphosphazene resin was mixed on a stirrer at 80 °C for 10 min to achieve a homogeneous mixture. The calculated amount of DDS was added to the matrix and stirred for 20 min at 125 °C till complete dissolution. In the Technical Data sheet of DDS it is recommended to dissolve the curing agent in epoxy matrix at 130 °C, however, systems could reach homogeneous state even at 125 °C. That is why we used 125 °C to avoid even a little possibility of precuring. Subsequent degassing of the system was performed at 125 °C for 15 min at a residual pressure of 1.0 kPa. It was found that pressure lower than 1.0 kPa at 125 °C is enough for the full degassing of the compositions. We used the pressure ≤1.0 kPa for quicker completion of degassing step. At the end of the degassing process, the resulting compositions were used as received for further curing study.

### 2.2. DSC Measurements

A Netzsch DCS 204 F1 Phoenix was used to monitor curing under isothermal conditions. The samples were encapsulated in aluminum pans. The instrument was calibrated with a standard metal calibration set under nitrogen purging at a 50 mL/min flow rate. The sample weight was kept in the range of 5–8 mg for all DSC runs.

The main factors that determine experimental conditions for the isothermal curing of thermosetting resins are the following:

1. How rapidly the system starts to cure under a certain isotherm.
2. How rapidly the DSC instrument reaches the dynamic and isothermal mode.

If the system begins to react actively until reaching the isotherm temperature, a significant amount of heat can be lost during the preheating section, resulting in incorrect enthalpy of cure. In the case of DSC inertial furnace, time to reach stabilized ramp or isotherm mode can be estimated in minutes, so preliminary heating step will dramatically increase. Thus according to the features of samples and DSC instruments, research utilizes one or the other method of carrying out isothermal runs [29]:

1. The most common way to carry out isothermal runs is a quick ramp to the isotherm temperature at a heating rate 50–100 °C/min. This mode is suitable for samples with low reactivity at temperatures below isotherm, in case of the inertial furnace of the DSC instrument. These conditions provide negligible loss of heat.
2. The next way for carrying out an isothermal experiment consists in heating the sample at a standard rate to the isotherm temperature. The main idea of this temperature program is that the significant heat released during dynamic heating is taken into account in further calculations of the total heat release. However, to follow such an experiment, it is necessary to have a DSC instrument with the shortest stabilizing time to reach the mode, otherwise, when passing from a dynamic segment to an isothermal one, there will be a large gap in the data.
3. The third type of isothermal measurement is the rapid insertion of a crucible with the sample into a preheated DSC cell. This version is suitable for low-temperature isotherms up to about 200 °C, limited by the technical characteristics of the instrument. It is suitable for those cases when the system is highly reactive, and the DSC has an inertial furnace and cell. So the time taken to reach the mode after introducing the sample into the preheated cell is much shorter than in the first case of quick temperature ramp to the isotherm temperature.

In our work the third type of carrying out the experiment was implemented.

The choice of the baseline is also an important point. In the case of the isotherm, all runs were carried out by applying baseline correction with temperature, sensitivity and Tau-R calibrations. When calculating the area under the curve, the baseline was chosen to be horizontal right started.

## 3. Results and Discussion

### 3.1. Carrying Out Experiments

In this study, we investigated quite reactive systems. All three ways of carrying out isothermal runs were tested.

For the first way we chose the following temperature program: ramp from RT to the isothermal temperature at a rate 50 °C/min followed by isothermal segment. In this variation of experiment, the time loss during preheating and stabilizing was about 4 min., which turned out to be significant enough for incorrect calculation of the full heat released during curing.

For the second way we followed the following temperature program: ramp from RT to the isothermal temperature at a rate 20 °C/min followed by isothermal segment. Calculated enthalpy during preheating was added to the enthalpy at isothermal run resulting in full heat released during curing. This method did not work due to the long stabilizing time at transition from the dynamic to the isothermal segment. The time taken was approximately 3 min.

The third way turned out to be the most reasonable from the point of view of obtaining a correct and complete DSC curve. The stabilization time of the DSC after opening the cell preheated to an isothermal temperature and inserting the crucible with the sample into it was about 1.5 min., which is significantly shorter than in all previous versions.

Thus, after this preparatory work all DSC runs were implemented according to the third way. For each composition five isothermal runs were made at temperatures of 150–200 °C (Table 2).

The enthalpies of all measurements are represented in Table 2. The increase in isothermal temperature results in the growth of the heat release due to a more complete cure. The presence of ECP in the composition leads to the decrease in enthalpy compared to unmodified system. The maximum values of enthalpy turned out to be 402, 411 and 362 J/g for formulations 1, 2 and 3 respectively. It is important to mention that there was no post-curing heat observed after a dynamic run of these samples, so no enthalpy was lost during estimation of maximum values.

The dramatic difference in enthalpy for formulation 3 could be explained by the enhanced concentration of ECP, which contains less epoxy groups than ED-20 and the enhanced stoichiometric hindrance due to the bulk molecule of ECP resulting in impossibility of complete cure. Moreover we assume that systems may undergo vitrification during curing. This statement is based on our previous studies of these systems, which showed glass transition temperatures to be higher than the curing temperature in some cases [17].

As one can see from Table 2, the values of enthalpies at the highest isotherm temperature for each formulation fall out of the dependency. They demonstrate dramatically low values of 356, 371 and 300 J/g for formulations 1, 2 and 3 respectively. We propose the following reasons for this feature. First of all there may be a significant error in the experiment as the temperature of the run is very high and much heat could be lost during DSC stabilizing. The second reason could be an incomplete cure that may occur due to very fast curing at a given temperature, resulting in an early vitrification and diffusion control that impedes the further curing process.

These experiments demonstrated that for compositions 1, 2 and 3 the maximum values of enthalpy appeared to be 402, 411 and 362 J/g respectively. However, we decided not to use these runs at high temperatures in kinetics study as for a more detailed investigation of this feature, an extra series of experiments at elevated temperatures must be implemented, that is beyond the scope of the present work.

### 3.2. Model Fitting

The DSC method makes it possible to trace structural transformations during curing both before and after gelation through the thermal effects accompanying these transformations.

The heat effects of curing and their nature depend on the curing mode. In Prime [30], Vyazovkin and Wight’s [31] studies, it was shown that the kinetic parameters of the process determined by the isothermal and non-isothermal methods for the same system could not completely correlate with each other. One of the main reasons is that isothermal and non-isothermal runs are inevitably carried out in different temperature ranges, where completely different effects and competitive reactions can be observed. However, nowadays the modeling of curing processes is actively developing, and some works [32] show the possibility of predicting with sufficient accuracy isothermal curing based on non-isothermal modeling. This accuracy is achieved due to the complication of mathematical calculations, the use of new calculation methods with peak separation [33], as well as carrying out extra measurements.

In practice, the process of curing epoxy resins is stepwise, including 2–3 isothermal segments. Therefore, the intention to bring the curing process closer to practice resulted in isothermal measurements in this study, rather than dynamic ones.

The absolute degree of conversion can be calculated:

(3) $$\beta =\frac{Q}{Q_{max}}$$

where *Q* is the heat released at certain isothermal temperature and *Q_max_* is the maximum heat of the process.

**Figure 2:**
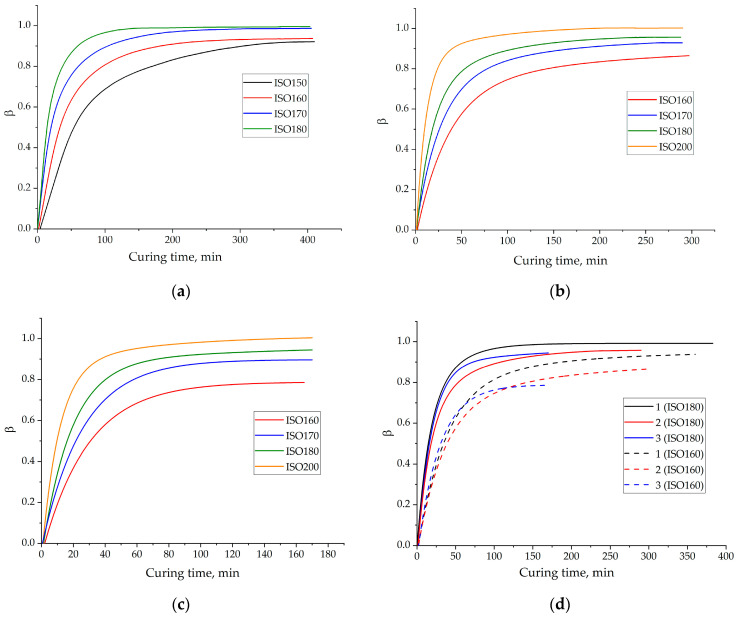
The dependences of the degree of conversion (*β*) on the curing time at isotherms: 150 (black), 160 (red), 170 (blue), 180 (green), 200 (orange) °C for formulation 1 (**a**), 2 (**b**), 3 (**c**). A comparison (**d**) of the *β*-dependencies for formulations 1 (black), 2 (red), 3 (blue) at isotherms 160 °C (dash), 180 °C (strait).

Figure 2 shows dependencies of the degree of conversion on the curing time, for the systems under study. 

Figure 2a–c demonstrate that all the curves have the same character. The decrease in isotherm temperature results in an incomplete cure that can reach, for example, even 0.77 degrees of conversion at 160 °C for formulation 3. Figure 2d shows that the higher temperature of isotherm applied less difference in the final degrees of conversion observed. The final degrees of conversion at 160 °C isotherms for formulation 1, 2 and 3 are 0.94, 0.86 and 0.77 respectively. The reduced values for modified compositions are observed due to incomplete cures resulting from bulkiness and the multifunctionality of the ECP molecule, which needs more energy for motion and bond formation compared to the unmodified system.

Applying a model fitting approach, the obtained dependencies were approximated by the 2-order Equation (4) until high degrees of conversion. This fitting appeared to be the most accurate compared with autocatalytic form of the 1-order model (6) [34] that is also typical for epoxy-amine curing (Table 3).

**Figure 3:**
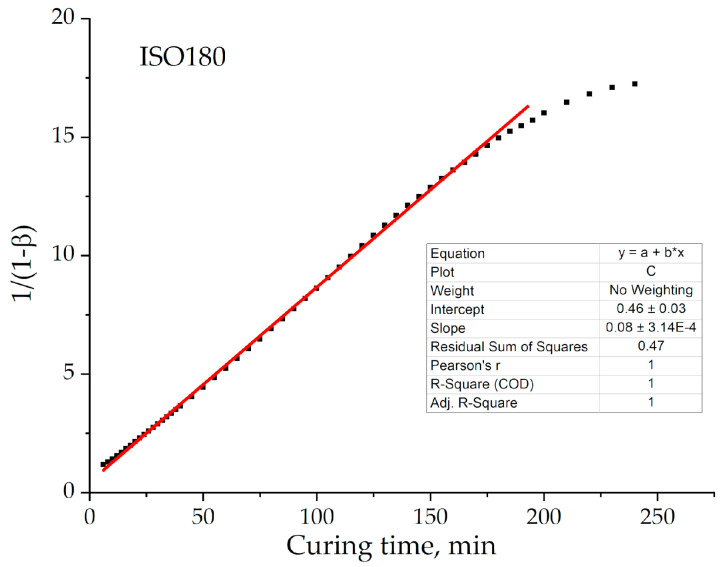
A graphical representation of the curing process in coordinates of the 2-order kinetic equation for formulation 2 of the isotherm of 180 °C, where the red line is the linear fitting and black square is experimental data.

Figure 3 shows graphical determination of the reaction rate constant as *tgα* of the linearized curve. This example (Figure 3) demonstrates that 2-order fitting is only applicable till high degrees of conversion *β* < 0.9. Thus, that is the point that change from a kinetic to a diffusion controlled mechanism may occur.

(4) $$\frac{d\beta }{d\tau }=k{\left(1\;-\;\beta \right)}^{2}$$

(5) $$\frac{1}{\left(1-\beta \right)}=k\tau +C$$

(6) $$\frac{d\beta }{d\tau }=\;k\left(1\;-\;\beta \right)\left(1+C\beta \right)$$

Table 4 presents the kinetic parameters of the modeling curing process with a 2-order equation for formulations 1–3: rate constants (k_2ord_) and activation energy (E_2ord_).

According to the rate constants (Table 4) it is clear that higher temperature leads to greater rate constants that corresponds to the acceleration of the process of curing.

**Figure 4:**
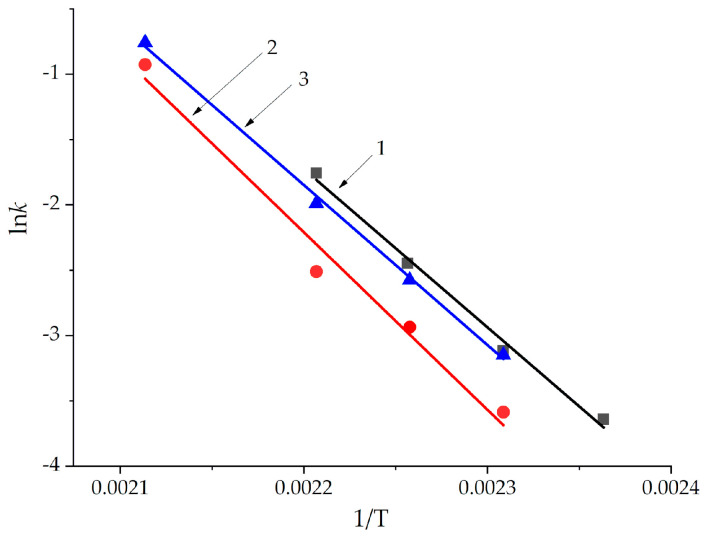
The dependencies of the rate constant logarithm (ln*k*) on the reciprocal temperature (1/T) for formulations 1 (black), 2 (red), 3 (blue).

Using the logarithmic form of the temperature dependence of constant (7), the activation energy was determined as tgα⋅*R* (Figure 4).

(7) $$k=k_{0}\;\cdot \exp\left(\frac{-E}{RT}\right)$$

The activation energies for all systems turned out to be approximately 100 kJ/mol (Table 4), which is slightly higher than that for ED-20 with DDS in literature [22].

**Figure 5:**
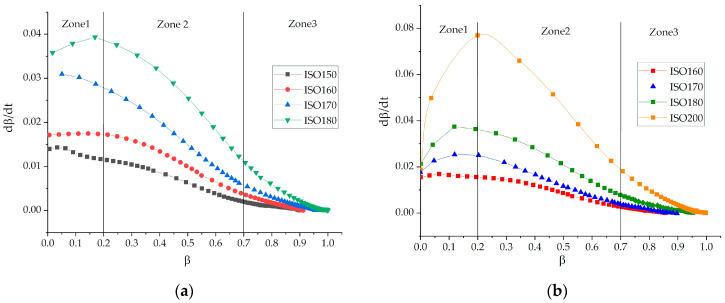
The curing rate-dependencies (d*β*/dt) on the degree of conversion (*β*) for formulations 1 (**a**) and 2 (**b**).

For more detailed consideration we represented curves in the form of d*β*/dt—t for n-order model fitting. The dependence of d*β*/dt—t is more sensitive than *β*—t. Figure 5 shows that we distinguished 3 zones and set critical transition points.

The critical conversions of transitions from one zone to the other appeared to be approximately 0.2 (zone 1–zone 2) and 0.7 (zone 2–zone 3). However, we admit that transition from one mechanism to another is not an instantaneous process, and often the characteristic transition points have a temperature dependence [19]. Zone 1 is characterized by the ambiguous behavior of the rates for all formulations. The duration of this stage is very small, being around 7 ± 5 min, and perhaps there could be some technical imprecisions that come out when representing data in a sensitive differential form. Therefore, it was difficult to apply any model to this first zone.

Zone 2 is characterized by the dramatic reduction of reaction rate as concentrations of initial components decrease and the viscosity of the systems increases. According to our previous rheological work [35] where we studied the process of epoxyphosphazene curing till gelation by viscometry, we matched the point of gelation with its degree of conversion. The results are presented in Table 5. The degree of conversion corresponding to gelation turned out to be exactly in the region of 0.7 ± 0.03 (Table 5). Thus, we found that the end of zone 2 correlates with physical process of gelation.

The general nature of the epoxy-amine curing is n-order. Therefore, the calculations for zone 2 were carried out according to Equation (8).

(8) $$\frac{d\beta }{d\tau }\;=\;k\cdot (1\;-\;\beta {)}^{n}$$

Zone 3 is characterized by incomplete curing, which is proved by the decrease in heat release of the reactions with decreasing isotherm temperature, so the maximum attainable degree of conversion is not achieved (Table 2). This zone was also calculated by the n-order reaction, but taking into account the incomplete curing according to Equation (9), where *β*_T_ is the maximum attainable degree of conversion at a given temperature.

(9) $$\frac{d\beta }{d\tau }\;=\;k\cdot (\beta_{T}\;-\;\beta {)}^{n}$$

**Figure 6:**
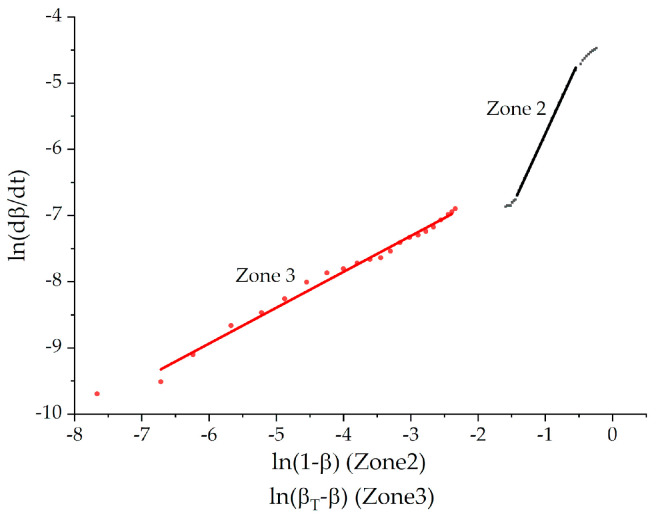
The process of curing in coordinates of the Equations (7) and (8) in logarithmic form for determining the reaction order (n) and rate constant (k) by linear fit for formulation 1 at isotherm of 150 °C.

The determination of the order and rate constant consisted in plotting the dependencies ln(d*β*/dt) − ln(1 − *β*) and ln(d*β*/dt) − ln(*β*_T_ − *β*) (Figure 6) of Equations (8) and (9) in logarithmic form, respectively, where the slope of the linearized section provided the reaction order, and the intercept with the ordinate axis provided the logarithm of the rate constant. The calculation results are presented in Table 6 and Table 7.

**Figure 7:**
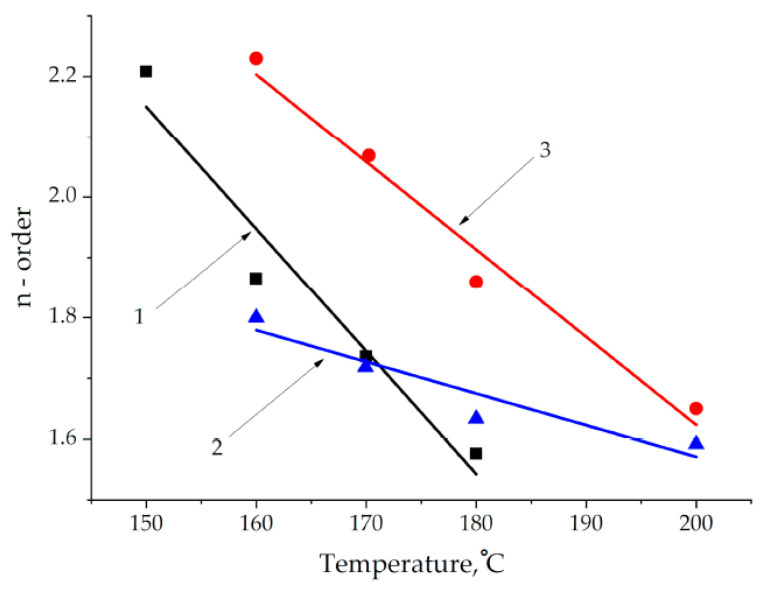
The reaction order-dependencies (n-order) on temperature for formulations 1–3.

Figure 6 represents an example of good linear fitting. The results of the calculations for zone 2 showed that the reaction order and rate constants are temperature-dependent parameters. Using the exponential equation for the temperature dependence of the rate constant (Equation (7)), the activation energy for zone 2 was calculated, which turned out to be quite close to the literature data for curing epoxy-amine binders (Table 7). The linearized temperature dependencies of the order are presented in Figure 7, and the resulting equations are presented in Table 6.

When adding epoxyposphazene to the composition, the reaction order turns out to be less dependent on the temperature compared to unmodified system.

The influence of epoxyphosphazene modifier in compositions on the kinetic parameters of curing is complex. The addition of 10 wt.% of ECP leads to the decrease of the rate constant from 0.1725 to 0.0813 min^−1^ and the increase of the activation energy from 100.2 to 112.0 kJ/mol compared with unmodified composition (Table 4). So we can say that 10 wt.% of ECP in the system slightly slows down the curing process, which is also proved by reduced degrees of conversion at the point of gelation for formulation 2 (Table 5). However, the addition of 20 wt.% of ECP returns the rate of curing to the level of unmodified composition.

When distinguishing zones and analyzing zone 2, which reflects the main stage of curing, it appeared that there is almost no change in rate constant; it increases by only ~0.004 min^−1^ compared to unmodified system as far as activation energy grows from 50.8 to 54.1 kJ/mol (Table 7). So for zone 2 we observe a tiny acceleration of the curing process when adding a modifier.

Zone 3 is well linearized using modified n-order equation for incomplete curing (9). In order to study the mechanism’s change during curing from zone 2 to zone 3, the isoconversional approach was applied.

### 3.3. Isoconversional Approach

If vitrification appears during curing, then there may be a change in the reaction mechanism to a diffusion-controlled one [36] that could be detected by changes in activation energy. We propose that our systems could vitrify during curing due to the fact that their glass transition temperatures are higher than the curing temperature in case of curing mode of 180 °C for 8 h being 182 °C and 202 °C for unmodified composition and with 20 wt.% ECP one respectively. These properties were described in our previous study of these systems dedicated to the physical characteristics [17]. Moreover, we know that incorporation of epoxyphosphazene increases the rate of viscosity growth during curing [35], and the degrees of conversion at the point of gelation slightly decrease during curing (Table 5). These factors could have some impact on mechanism’s shift to the region of diffusion control.

In this regard, the use of isoconversional methods is relevant in this work, as it makes it possible to determine the activation energy over the entire range of conversion degrees.

According to the basic principle of the isoconversional approach, the reaction rate at a constant degree of conversion is only a function of temperature (10). This statement is fulfilled when f(β) is independent of temperature, which in turn is possible only in a single-step process. If the process is complex, then the relative contribution of the stages will be reflected in the values of the activation energy, caused by ambiguous determination of the degrees of conversion.

Determination of the activation energies at different degrees of curing by the Friedman method [21] consisted in plotting dependence 11 of the Equation (2) in logarithmic form (12), where the slope of linearized curve provided $-\frac{E}{R}$.

(10) $$\frac{d\ln{\left(\frac{d\beta }{dt}\right)}_{\beta }}{dT^{-1}}=\;\frac{-E_{\beta }}{R}$$

(11) $$\ln{\left(\frac{d\beta }{dt}\right)}_{\beta }-\;\frac{1}{T}$$

(12) $$\ln{\left(\frac{d\beta }{dt}\right)}_{\beta }=\ln\left[Af\left(\beta \right)\right]-\frac{E}{RT}$$

**Figure 8:**
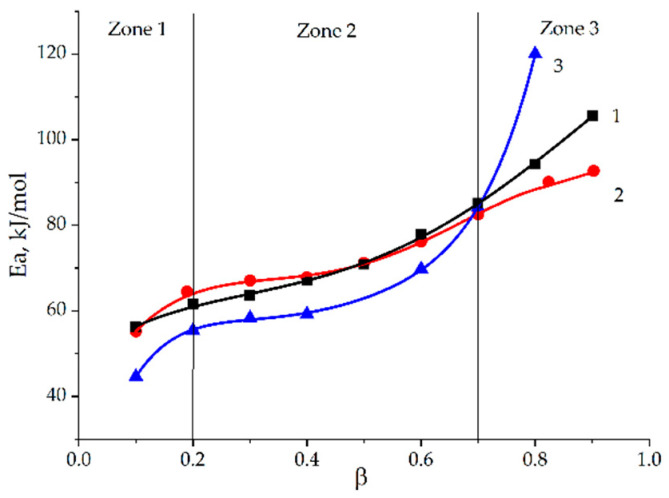
The Ea-dependencies (Ea) on the degree of conversion (*β*) according to the Friedman method for formulations 1–3.

The calculation results are shown in Figure 8. It is interesting that the obtained dependencies also show three characteristic areas with similar characteristics of transitions: zone 1–zone 2 *β* = 0.2, zone 2–zone 3 *β* = 0.7. The activation energy is not constant during curing; it grows with the increase in the degree of conversion. It determines the complex nature of curing that often results in the multistage mechanism of the total cure.

The changes of activation energy in the final stages are usually assigned to diffusion contributions [21,37].

The maximum value of the degree of curing does not depend on the temperature and is equal to 1 (*β*_max_ = 1). When curing at temperatures below the maximum attainable glass transition temperature for a given system, the system can undergo vitrification at a certain final stage of curing. As a result, the maximum degree of conversion at a certain isotherm turns out to be less than 1 (*β*_T_ < 1).

When calculating the activation energy by the isoconversional approach, Vyazovkin [21] proposed the use of relative degree of conversion that is determined as follows:

(13) $$\beta^{\prime }=\frac{\beta }{\beta_{T}}$$

**Figure 9:**
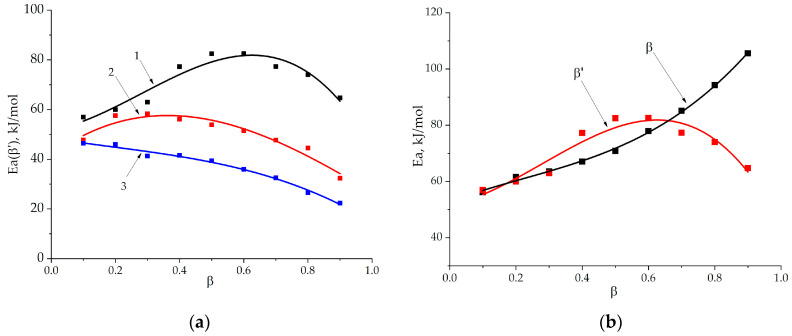
The Ea-dependencies (Ea) on the relative degree of conversion (*β*’) calculated by the Friedman method for formulations 1–3 (**a**). A comparison of Ea-dependencies calculated by the Friedman method for formulation 1 using absolute (*β*) and relative (*β*’) degrees of conversion (**b**).

We applied this approach of relative degrees of conversion and recalculated activation energy by the Friedman method (Figure 9a,b).

Figure 9 shows that application of the absolute degrees of conversion for incompletely cured systems gives overestimated values of the activation energy at the final stage of curing. Formulation 1 has the maximum values of activation energy (80 kJ/mol) followed by formulation 2 and 3. For all systems activation energy decreases during curing, having only a slight maximum for formulation 1 and 2. The proposed diffusion control mechanism at the final stage of curing in zone 3 is in good agreement with the literature data that a change in the nature of the reaction to a diffusion-controlled one is characterized by a decrease in the activation energy values [21,37,38]. Thus, if the increase in ECP concentration in the system provides the reduction of activation energy values, especially at final stage, we could propose that modification with epoxyphosphazene shifts the curing process towards the diffusion-controlled region.

### 3.4. Diffusion-Controlled Region

For brief approval or disapproval of proposed diffusion control, we applied the following equation suggested by A.Ya. Malkin and V.G. Kulichikhin to describe the macrokinetics of a self-retarding process [39].

(14) $$\frac{d\beta }{dt}=k\left(1-\beta \right)\left(1-\xi \beta \right)$$

where *ξ* is a dimensionless parameter accounting for the self-retardation effect in the late stages of the reaction.

(15) $$\xi =\frac{1}{\beta_{T}^{max}}$$

where $\beta_{T}^{max}$ is the limiting conversion reachable at the given curing temperature. It is obvious that at *ξ* = 1 Equation (14) transforms to a 2-order kinetic equation that is applicable for the systems under study.

**Figure 10:**
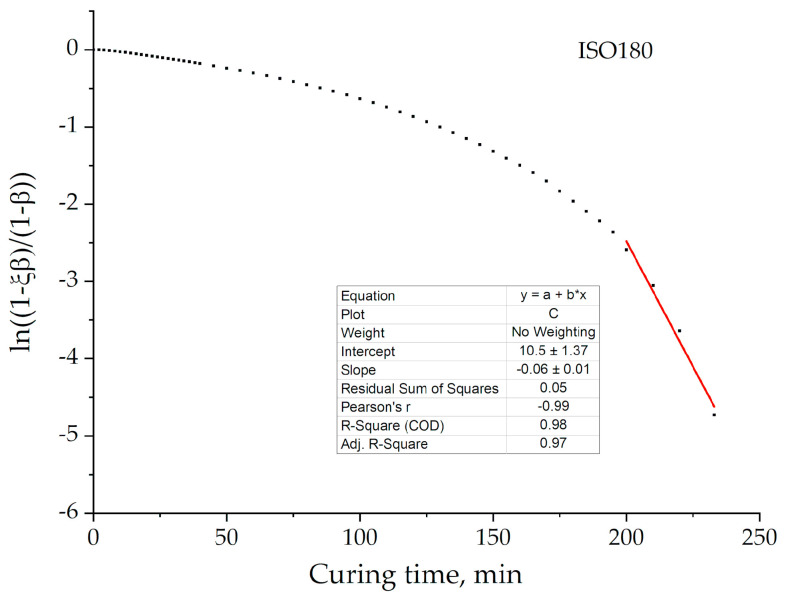
A graphical representation of the curing process in coordinates of the 1-order kinetic equation with self-retardation for formulation 2 at an isotherm of 180 °C, where the red line is the linear fitting and black dots are experimental data.

The graphical plotting of dependences *β*(t) in the coordinates [ln(1 – *ξβ*)/(1 – *β*)] – t confirms the applicability of the self-retarded process for our systems. Figure 10 presents the example of this plotting for formulation 2 at 180 °C. Figure 3 and Figure 10 demonstrate that 2-order model is replaced by the 1-order self-retarded one at a value of conversion approximately equal to 0.94 (~200 min).

The critical points of transition from one mechanism to another correspond to rather high conversions (>0.9), which are far behind the gelation (~0.7). Thus, it is necessary to find another physical contribution to this mechanism’s change; that is, to determine vitrification during curing and glass transition dependency upon the degree of conversion. We intend to obtain these experimental data in our further study to give a more accurate model for the final stage of curing, applying for example the well-known Rabinowitch model [40].

## 4. Conclusions

This study revealed the influence of epoxyphosphazene modifier on the isothermal process of epoxy-amine curing by means of model fitting and the isoconversional approach. Under the model fitting approach we detected the n-order nature of the reaction for all systems under study. In particular, the 2-order equation fits well the main part of curing, excluding final stage with high degrees of conversion. The order turned out to be a temperature dependent parameter. The addition of 10 wt.% of ECP makes the order less dependent on temperature.

The nature of epoxyphospazene impact on the rate constant is complex depending on the method. When we apply the 2-order equation, incorporation of 10 wt.% of ECP leads to a slight decrease in the rate constant. However, when we apply an n-order model to zone 2, the rate constant slightly increases. However, it should be mentioned that all these changes are almost negligible. These results proved our proposal in the work [35] that the main reason for the acceleration of viscosity increase during curing is the multifunctionality of the modifier and bulkiness of its molecule, but not the acceleration of the process of curing, as the rate constants do not increase monotonously with addition of ECP (Table 4).

We figured out three zones during curing by applying both model fitting an thed isoconversional approach. The critical transition points appeared to be approximately the same for all compositions. They are *β* = 0.2 for zone 1 to zone 2 and *β* = 0.7 for zone 2 to zone 3. It is of interest that transition from zone 2 to zone 3 correlates with physical process of gelation.

Under isoconversional study of activation energy during curing by the Friedman method, we defined the difference between calculations in absolute and relative degrees of conversion. Absolute values provide the rise of activation energy for all systems, while relative ones provide the decrease. The addition of epoxyphosphazene to compositions influences the activation energy, reducing its values, and the reduction of Ea values at the final stage of curing usually corresponds to the diffusion-controlled mechanism. The self-retarded model by A.Ya. Malkin and V.G. Kulichikhin was probed, and appeared to be applicable at high degrees of conversion.

The study revealed some features of epoxy curing with ECP applying hot curing agent (DDS). They differ much from conclusions made in our previous kinetic work upon the same epoxyphosphazene binders but with another curing agent—L-20 (cold curing) [36]. The objects of studies have different kinetic descriptions during the first part of curing: a 2-order equation and 1-order autocatalytic one. In the present work the diffusion-controlled region definitely appears later than in previous study. We can definitely state that the hot curing process (with DDS) provides more complete curing of the compositions in comparison with cold curing (with L-20).

## Figures and Tables

**Table 1:** The formulation of the mixtures.

Formulation Number	Component Content, wt.%	
	ED-20 ^1^	ECP ^2^
1	100	0
2	90	10
3	80	20

^1^ ED-20—Bifunctional bisphenol-A-based epoxy resin of ED-20 brand; ^2^ ECP—epoxycyclophosphazene oligomer.

**Table 2:** The enthalpies of curing for isotherms at 150, 160, 170, 180, 200 °C for formulations 1–3.

Formulation Number	Q_T_, J/g					
	150 °C	160 °C	170 °C	180 °C	200 °C	220 °C
1	365	374	392	402	356	-
2	-	358	381	393	411	371
3	-	288	324	345	362	300

**Table 3:** The values of R2 according to the 2-order equation for formulations 1–3.

Formulation Number	Model	R2				
		150 °C	160 °C	170 °C	180 °C	200 °C
1	2-order	1	1	0.98	0.95	-
2		-	1	1	1	0.96
3		-	1	1	1	0.97
1	Autocatalytic 1-order	0.94	0.89	0.96	0.91	-
2		-	0.88	0.90	0.94	0.97
3		-	0.92	0.94	0.94	0.99

**Table 4:** The values of the rate constants (k_2ord_) and activation energies (E_2ord_) according to the 2-order equation for formulations 1–3.

Formulation Number	k_2ord_, Min^−1^					E_2ord_, kJ/mol
	150 °C	160 °C	170 °C	180 °C	200 °C	
1	0.0262	0.0443	0.0864	0.1725	-	100.2
2	-	0.0277	0.0531	0.0813	0.3963	112.0
3	-	0.0428	0.0765	0.1368	0.4685	102.6

**Table 5:** The degrees of conversion at the points of gelation for formulations 1–3.

Formulation Number	*β*_gel_ at the Temperature of (°C)		
	160	170	180
1	0.71	0.72	0.73
2	0.66	0.67	0.68
3	0.68	0.69	0.70

**Table 6:** The reaction order and rate constants of zone 2 for formulations 1–3.

Formulation Number	n-Order	Temperature Dependence of n-Order
1	2.21–1.57	n = 5.19 − 0.02⋅T
2	2.23–1.65	n = 4.52 − 0.015⋅T
3	1.80–1.59	n = 2.61 − 0.005⋅T

**Table 7:** The values of rate constant (k_n-ord_) and activation energy (E_n-ord_) of zone 2 for compositions 1–3.

Formulation Number	k_n-ord_, Min^−1^					E_n-ord_, kJ/mol
	150 °C	160 °C	170 °C	180 °C	200 °C	
1	0.02845	0.03538	0.04811	0.07467	-	50.8
2	-	0.03995	0.05522	0.07445	0.13923	53.1
3	-	0.03696	0.05261	0.07499	0.13155	54.1

## Data Availability

The data presented in this study are available on request from the corresponding author.
